# Effects of anti-TNF therapy on ophtalmological complications in children with rheumatic diseases

**DOI:** 10.1186/1546-0096-12-S1-P236

**Published:** 2014-09-17

**Authors:** Marijan Frkovic, Nenad Vukojevic, Marija Jelusic

**Affiliations:** 1 Division of Paediatric Rheumatology and Immunology, University Hospital Centre Zagreb, Zagreb, Croatia; 2 Departement of Ophtalmology, University Hospital Centre Zagreb, Zagreb, Croatia

## Introduction

Biologics started a new era in treatment of different aspects of rheumatic disases. Authors present the effects of anti-TNF therapy on ophtalmological complications in their patients with juvenile idiopathic artritis (JIA), juvenile dermatomiositis (JDM) and sarcoidosis.

## Objectives

To investigate therapeutical sucess of anti-TNF therapy on ophtalmological complications in children with rheumatic diseases.

## Methods

Retrospective chart rewiev of all rheumatic patients with ophtalmological complications treated with anti-TNF therapy at Division of Paediatric Rheumatology and Immunology, University Hospital Centre Zagreb, during 2009. – 2013. period.

## Results

Among 54 children treated with anti-TNF therapy at our Division during 2009. – 2013. period, 10 were detected with ophtalmologycal complications (8 girls, 2 boys), 8 with JIA, 1 with JDM and 1 with sarcoidosis. Nine patients had chronic uveitis (JIA, sarcoidosis) and 1 had …cotton wool“ retinal lesions accompanied with bilateral papilar oedema (JDM). Avarage time between the begining of disease and start of anti-TNF therapy was 2.9 years (28 days – 9 years). Initial anti-TNF therapy was adalimumab in 5 cases (4 JIA, 1 sarcoidosis), etanercept in 4 cases (JIA) and infliximab in 1 case (JDM). During therapy, etanercept was changed to adalimumab in 2 cases, due to inefficacy on uveitis. Adalimumab was partially effective in 1 case (sarcoidosis). Infliximab, applied in patient with JDM and …cotton wool“ retinal lesions accompanied with bilateral papilar oedema, led to fast and complete recovery of eye changes and other aspects of the disease.

## Conclusion

Anti-TNF therapy was successfull in 90 % of our cases. To our opinion, anti-TNF therapy should be early introduced in rheumatic patients with severe ophtalmological complications.

## Disclosure of interest

None declared.

